# Assistive Technologies and Quadriplegia: A Map Point on the Development and Spread of the Tongue Barbell Piercing

**DOI:** 10.3390/healthcare11010101

**Published:** 2022-12-28

**Authors:** Antonia Pirrera, Paola Meli, Alessandra De Dominicis, Alessandra Lepri, Daniele Giansanti

**Affiliations:** 1 Centro TISP, Istituto Superiore di Sanità, 00161 Roma, Italy; 2 FARVA, Istituto Superiore di Sanità, 00161 Roma, Italy

**Keywords:** spinal cord injuries, assistive technology, tongue barbell piercing, tongue drive system, inductive tongue control system

## Abstract

The barbell piercing can be used as an assistive device that allows people with severe disabilities, such as tetraplegia, to control their environments using the movement of the tongue. The human tongue can move rapidly and accurately, such that the tip can touch every tooth. Lingual control systems allow people with disabilities to take advantage of their residual skills for easier communication and to improve the control of mobility and the surrounding environment. The aim of this study was to conduct a narrative review of the development and dissemination of the assistive technologies based on tongue control by means of the barbell piercing. The design of the study was based on: (I) an overview of Pubmed complemented with other databases and Web searches (also institutional); (II) an organization according to a standardized checklist for narrative reviews; (III) an arrangement with four different perspectives: the trends in the scientific literature, technological evolution and categorization, dominant approaches, issues of incorporation into the *health domain*—such as acceptance, safety, and regulations. The results have highlighted: (1) that the volume of scientific productions, which started in this sector before the smartphone expansion, has not increased; (2) that it is possible to make a map point of the technological evolution and categorization; (3) that these assistive technologies have a high degree of acceptance and performance, especially when integrated with aid tools with mechatronics; (4) and the complexity of the regulatory framework in this area. The study, from a general point of view, highlighted the high potential of these systems and we suggest investing the energy into agreement tools for assistive technologies (AT)s, such as health technology assessment studies, comparative assessment analysis, or consensus conferences that could allow a better diffusion and use of ATs, including these systems.

## 1. Introduction

This study moves into the sector of severe motor disabilities. Among these disabilities, quadriplegia has an important impact on the autonomy and quality of life of a person. Assistive technologies have enormous potential for supporting the communicative functions of people with tetraplegia and are therefore the theme of important studies relating to engineering developments and clinical applications. Among the assistive technologies used in quadriplegia, it is also possible to find some technologies based on the barbell piercing.

### 1.1. Spinal Cord Injuries and Tetraplegia: Definition

A spinal cord injury is the result of damage to any portion of the spinal cord or the nerves at the base of the spine. The spinal cord is a bundle of nerve fibers and tissue, which lies within the spine forming the brain’s connection to the body.

Damage to any part of the spinal cord can affect sensory, motor, and reflex capabilities if the brain is unable to send information past the location of the injury. 

A severe spinal cord injury (SCI) or traumatic brain injury (TBI) can have a variety of impacts [1]. Patients of SCIs and TBIs can suffer a certain type of constant paralysis. An SCI can be complete (totally affecting the spinal cord) or incomplete (partially affecting the spinal cord). Furthermore, it should be considered that the higher the damage is positioned on the spine, the more serious the paralysis will be. We must consider that the spine is divided into four districts: cervical (C1–C7, C8*), thoracic (T1–T12), lumbar (L1–L5), and sacral (S1–S5) (*there is an additional cervical-level injury known as a C8 injury, which relates to damage to the spinal cord root that exits the spinal column between vertebrae C7 and T1). Tetraplegia refers to damage in the cervical district.

The simplest Tetraplegia definition [1] is “a form of paralysis that affects both arms and both legs “This term is often replaced with the term “Quadriplegia”. Table 1 reports the neuromotor limitations as a function of the location of the damage [1]. The level of individual disability depends on several factors, such as the position of the injury, its completeness, and the timeliness of the treatment of the injury. Therefore, the technologies must be properly planned and assigned to support the disability considering this.

### 1.2. The Assistive Technologies and the Tetraplegia

The WHO has addressed the issue of Assistive Technologies (ATs) and reports on its website [2] as they are a fundamental instrument of equity, independence, and dignity. In other words (literal quote),

*“Assistive technology enables people to live healthy, productive, independent, and dignified lives, and to participate in education, the labour market and civic life. Assistive technology reduces the need for formal health and support services, long-term care and the work of caregivers. Without assistive technology, people are often excluded, isolated, and locked into poverty, thereby increasing the impact of disease and disability on a person, their family, and society.”*

According to the Assistive Technology Industry Association (ATIA), an AT is any item, piece of equipment, software program, or product system that is used to increase, maintain, or improve the functional capabilities of persons with disabilities [3]. An AT supports people who have difficulty speaking, typing, writing, remembering, pointing, seeing, hearing, learning, walking, and many other things.

These aids are complex devices that often use special materials and complex mechanics. They are controlled by high-level electronics and information technology (e.g., motorized exoskeletons). In the case of aids for supporting the communication capabilities of people with communication disabilities, so-called devices for “alternative and augmentative communication” (e.g., eye pointers) are used [4]. The ATs for quadriplegia have been greatly affected, as for other forms of disability, by the exceptional technological developments of recent decades, and, more specifically, by the miniaturization of electronics, and the diffusion and integration into the *health domain* of mobile technology, micromechanics, and materials. These ATs allow people with tetraplegia both to interact with the environment and to communicate. They allow for the control of your smartphone, tablet, computer, wheelchair, robotic devices, and more, usually from one singular controller/device. This gives the person control over the Ambient Assisted Living systems for domotics, communication, and displacement.

When quadriplegia is addressed, it is necessary to carefully consider both the different forms of neuromotor disability and other aspects such as age, literacy, and the presence of any cognitive disorders, while also considering other factors such as the patient’s ability to accept a certain assistive technology. In this context, it is important to adapt the ATs to the person [5].

All of this is also in compliance with the latest document, the “International Classification of Functioning, Disability and Health” [6], which no longer looks at disabilities but at the *health components* of the person.

### 1.3. The Barbell Piercing as an Assistive Technology

In recent years, among the various AT solutions that have been developed to support tetraplegia patients with environmental control, communication, and displacement, it is possible to find those based on barbell piercing. Body piercing is defined as the penetration of an ornament into openings made in the skin or mucosa. Intraoral and perioral sites are often selected for piercing with the tongue, lips, and cheeks being the most pierced sites.

The spread of “body art” practices has given rise to increasing concerns. 

We previously performed studies and analyses on tattoos and piercings designed to improve the lives of those who suffer from particular diseases [7]. Medical tattoos applied to restore the bodily integrity of cancer patients are an example. 

There is also a great interest in the application of piercing in assistive devices, based on tongue control systems that can help to improve the autonomy of people with severe motor disabilities.

The barbell piercing can also be used as an assistive device that allows people with severe disabilities to control the environment using the movement of the tongue. The human tongue can move rapidly and accurately and the tip can touch every tooth. Lingual control systems allow people with disabilities to take advantage of their residual skills for easier communication and to improve the control of mobility and the surrounding environment. 

Regarding the sensor in these ATs, an activation unit made of a soft ferromagnetic material magnet can be used to carry out actions based on distance or support with appropriate devices.

Therefore, such an activation unit placed, for example with adhesive, on the tongue can interact with other receiving devices internally or externally from the mouth.

The use of a piercing as an alternative solution guarantees better stability, as the actuator is firmly anchored on the tongue through it. The positioning of the actuator by means of an adhesive solution presents less stability at the price of easier removal.

A technological study proposed at the Micro and Nanotechnology Sensors, Systems, and Applications IX congress in 2017 [8], highlighted the technologies used, and the classification of the devises used in this field. 

### 1.4. Purpose, Organization and Key Questions to Answer

The main objectives of this study are to:

- Investigate the evolution of these devices; the volume of scientific production; describe the use of tongue piercing as a driving tool for AT systems in quadriplegic patients enrolled in the study protocols.
- Analyze the use of these devices and their integration into the *health domain*.

The work is organized into five sections plus the introduction (*section one*).

*Section two* is the methods.

*Section three* reports the results. It is divided into *four parts*:

- The *first part* analyses the scientific production in this area, highlighting the progress and the evolutions.

This part answers the key question: “*How has scientific production evolved in this area?*

- The *second part* reports the technological evolution and categorization of the tongue-based devices.

This part answers the key question: “*What are the technological evolutions of these devices and how can they be categorized?*”

- The third part, reports the consolidated approaches using the barbell piercing.

It answers the key question “*What are the dominant approaches regarding the use of the barbell piercing inside the mouth?*”

- The *fourth part* analyses the integration into the *health domain* with a particular focus on the acceptance, safety, comparison, and regulation issues. It answers the heterogeneous question “*What can be said about the acceptance, comparison, safety, and regulation of these devices?*”

*Section four* reports a discussion on the evidence gathered also based on the deployment, and the development prospects.

*Section five* is dedicated to the conclusions.

## 2. Methods

This study made use of a standardized checklist for narrative reviews (Available online: [9]).

Given that this study investigated the integration in the *health domain*, the search was conducted on Pubmed. The search was also complemented, when necessary based on the topic addressed (for example legislation), by deepening the search using other databases dedicated to technological studies, and websites, including institutional ones. Tables 2 and 4 show the search terms used in the relevant sections. 

The design of the study, in line with the objectives, addressed four specific points of view with targeted searches to give the reader a complementary image from multiple angles and perspectives on specific aspects. The first search was dedicated to analyzing the trend of scientific production. The second search addressed the technological evolutions and the categorization of the devices. The third search was dedicated to the dominant approaches in the development of the sensor–actuator chains inside the mouth. The fourth search addressed aspects related to integration into the *health domain,* such as acceptance, comparison, security, and regulatory aspects.

## 3. Results

### 3.1. The Development of the Tongue Piercing as a Driver for Assistive Technologies in the Scientific Literature

In line with the purpose of the study, we:

- Turned to the PubMed database to analyze the evolution of scientific production in this area in relation to integration into the *health domain*.
- Defined a search key suitable for the purpose.
- Checked for any reviews.

The composite key is available and can be found in Table 2 [10].

The *first piece of general evidence* to be noted is that the list did not show the presence of reviews. This is certainly a comforting finding for our study in relation to the need to make a map point.

The *second piece of general evidence* is that the search returned 72 contributes, starting from the year 2006 up to the date of this review (the year 2022), a span of about 16 years. This means that the spread of these systems began before the boom of smartphones, as we know them today [11], and followed its evolution. With a few exceptions, regarding studies dealing with the biocompatibility of materials and lingual models (and the related issues), all the studies are focused on this area.

The *third general* consideration concerns the progress of scientific production.

**Figure 1:**
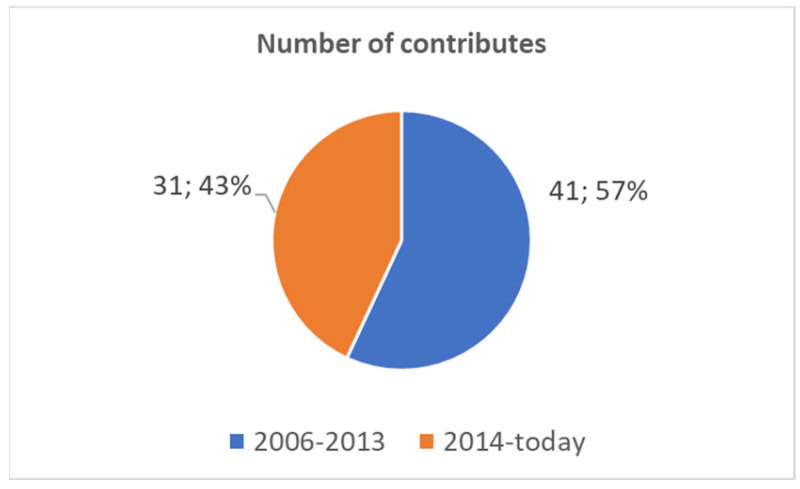
Scientific production for tongue electronic systems.

The peak of scientific production was in 2012 with 10 papers. In the first 5–6 years, up until the year 2012, the year in which the maximum number of studies, 10, was proposed, there was a growth in production. After this period, the trend changed. Figure 1 shows how: (a) up to the year 2013, approximately the first half-period of the scientific production period, we had 41 papers (57% of scientific production). (b) From the year 2014 up to today, approximately the second half-period of the scientific production period, we have had 31 papers (43% of scientific production). Twenty-five (34.7 %) of the total scientific works are contributions to International Congresses.

By addressing the scientific aspects, it is possible to see that the history and evolution of this technology are due to two international groups, which, starting from the year 2006, through initial contributions in [12,13], contributed to the theme with a different approach. In fact, with a few isolated exceptions [14,15], the scientific contributions were made by these scholars.

The technological approaches have evolved and adapted from time to time into different applications for the *health domain* and have shown important potential for integration with Alternative and Augmentative Communication, for integration with the environment (Ambient Assisted Living), and other home automation applications [16,17,18,19,20,21]. 

These two intraoral technological approaches produced through the two groups, in their different versions, integrations, and evolutions, demonstrated potential in the field of assistive technologies. 

### 3.2. Applied Technologies and Categorization

In order to analyze the applied technologies and approaches in this area, the search was integrated with other technology-focused databases. Some scholars reported in [8] an interesting categorization useful as a reference. We briefly report the technological evolutions and approaches according to this categorization also resumed in Table 3.

#### 3.2.1. Tongue Drive System

The first device, the Tongue Drive System (TDS) [8], used a magnetic field generated by a magnetic actuator-tracer on the patient’s tongue to detect instructions based on a set of tongue-based gestures [22]. The authors designed a headset with a pair of lateral poles arranged with sensors to detect and measure the variation of the magnetic field, correlated to the tongue-based gestures. The detectors were four tri-axial magnetic sensors. The communication to the receiver was wireless. The authors used a Texas Instrument device. The frequency was set at 2.4 GHZ [23,24,25]. The interface used the software Labview for both the A/D conversion and display [26].

#### 3.2.2. Intraoral Tongue Drive System

An evolution of the TDS [8,27,28,29] considered the placement of the receiver in the mouth. This new architectural design improved the detection of the magnetic field correlated to the tongue-based gestures, as the detectors were closer to the actuator-tracer placed on the tongue [30]. The receiver was anchored on the teeth. This allowed for better steadiness and discretion (with the system being hidden inside the mouth) [31]. Two evolutions were proposed. The first version used a custom system-on-chip device placed in the center of the device’s printed circuit board (PCB). Four 3-axial magnetic sensors were affixed on the four corners of the PCB. The system used a dual band (27 MHZ, 433 MHZ). The system communicated the data similarly to the previous system. The second version [32] proposed an arch-shape device, placed on the lower jaw, with the electronics located in the buccal shelf area. The frequency used was the same. The reception on the receiver side was improved using a super-regenerative approach.

#### 3.2.3. Standalone Tongue Drive System

The design of a device that could fit completely inside the mouth, i.e., a standalone device, was the next step [8]. The first device used a common open-source miniaturized platform, named Beagle Bone Black (BBB) [8]. The calibration based on an ARM A8 processor included a classifier designed by means of a support vector machine. This system allowed for successful interfacing with an electronically driven wheelchair [33]. Another device included a field programmable gate array (FPGA). The FPGA collected digitized raw information from the detectors using a serial peripheral interface [34,35]. A properly designed classifier algorithm based on the Earth’s magnetic field attenuation and logistic regression was implemented in the FPGA. Bluetooth low energy (BLE) was also used to interface with the pc or the smartphone.

#### 3.2.4. Multimodal Tongue Drive System

The integration of the TDS with further assistive technologies has been proposed [8]. The authors named it the Multimodal Tongue Drive System [36]. It integrated a head tracker and a speech recognition system [36]. The head tracker used an Inertial Measurement Unit comprising both one 3D gyroscope and one 3D accelerometer. The Dragon Naturally Speaking tool was used as an interpreter/speech-to-text translator. 

#### 3.2.5. The Inductive Tongue Control System

A further device used inductive sensors [8,37,38,39,40,41]. It was proposed at Alborg University in Denmark. It comprised: (a) a ferromagnetic actuator-tracer placed on the tongue; and (b) a group of 18 inductive switches placed on a PCB designed to be affixed on the palate. When the actuator touches the switches, the result is a voltage change and therefore a command. We will return to this later in the paper (see Section 3.3).

#### 3.2.6. Further Tongue-Based Systems

Further approaches [8] in progress are using light-emitting diodes and light detectors [42], the IBM tongue-track-point technologies [43], piezoelectric sensors [44], and a tongue-based Joystick (Jouse3) [45]. 

### 3.3. The Tongue Piercing in Assistive Technologies in Quadriplegia Today: The Two Most Important Approaches

Different technological approaches have been followed in the use of assistive systems based on the movement of the tongue. It was possible to carry out the categorization shown in Table 4. Surely, future efforts will be directed towards the bringing of all the ICT inside the mouth, and pushing towards miniaturization, towards greater comfort of the Stand-Alone Tongue drive system (*Position 3*, Table 3), and towards the expansion of Multimodal Tongue Drive Systems (*Position 4*, Table 3).

Looking from another perspective, however complementary, in line with the objectives of the study, which intends to address the processes of inserting the barbell piercing into these systems, we can focus on the Tongue Drive System and its intraoral evolution (*Position 1-2*, Table 3) and on the Inductive Tongue-Based System (*Position 5*, Table 3). In fact, two different approaches [12,13] have been proposed by scholars in the past, evolving in the recent two decades that use the movement and the multiple capabilities of the tongue, providing a specific tongue cockpit, which led to the insertion of the barbell piercing. One approach is the Tongue Drive System device, also available in the intraoral version (iTDS). Another approach is the Inductive Tongue Control System (Itongue). A description of the functioning and of the evolution of the two systems is provided below.

#### 3.3.1. The Tongue Drive System (TDS) in detail 

**Figure 2:**
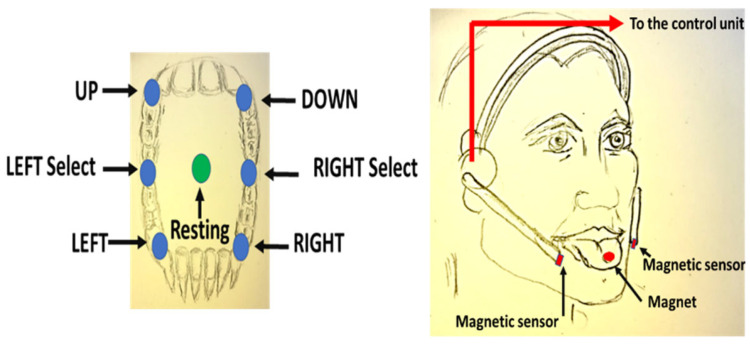
Sketch (realized by D.G.) of the first version of the TDS.

**Figure 3:**
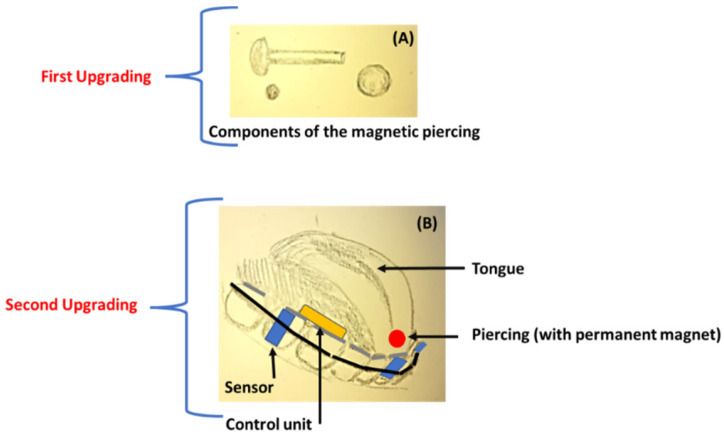
Sketch (realized by D.G.) of the upgrade to the TDS: (**A**) the introduction of the magnetic piercing; (**B**) the introduction of the oral control unit.

The TDS designed and developed initially at the North Carolina State University is a wireless and portable human–computer interface. The device consists of a small magnetic disk, attached by means of a dental adhesive onto the tongue, capable of generating a magnetic field [13]. The movements of the tongue induce variations in the magnetic field, which are detected using a kit of sensors positioned on an earphone. The signals are then sent wirelessly from a control unit, placed in the headset, to a receiver that processes them and translates each movement into a specific function defined by the user, such as moving the cursor on the screen of a computer, dialing a phone number, driving a wheelchair, or turning the light on and off. The TDS was capable of detecting six positions in the mouth, which are activated when reached by the tongue and translated into six commands. The TDS featured several important upgrades. Figure 2 shows a sketch of the first release. We report here two *important upgrades* (see in Figure 3 the sketch of the two upgrades).

The *first important upgrade* in the year 2014 consisted of inserting a magnet inside a barbell piercing to maintain the greatest level of efficiency over time. The magnet that generates the magnetic field was placed inside the dorsal sphere of a titanium barbell piercing, internally drawn, to which, after insertion into the tongue, a retainer is screwed in the ventral position, like a common lingual piercing. A medically performed tongue-piercing method was developed and tested for use with the TDS by people with high-level SCIs [46,47]. The piercing attached to the magnetic disc with adhesive has different implications due to the stable implant inserted into a body part (the tongue). The adhesive disk is not a stable device; however, it is useful for running some proof-of-concept experiments. The patients involved in the study evaluated the TDS as effective in interfacing with computers, driving wheelchairs, dialing telephone numbers, and other tasks, as described later in the paper. This device turned out to be even easier to use than the sip-n-puff device, as described later in the paper (see Section 3.4). 

*The second important upgrade* eliminated the headset. In this device, named Internal TDS (iTDS), the control unit was inserted into an apparatus similar to an orthodontic appliance (Figure. 3). The use of the iTDS by patients showed better compliance in comparison with the TDS, due to the absence of the earpiece [34].

#### 3.3.2. The Inductive Tongue Control System (Itongue) in Details

The Itongue was developed at the Center for Sensory–Motor Interaction at Aalborg University in Denmark [39,48,49,50].

**Figure 4:**
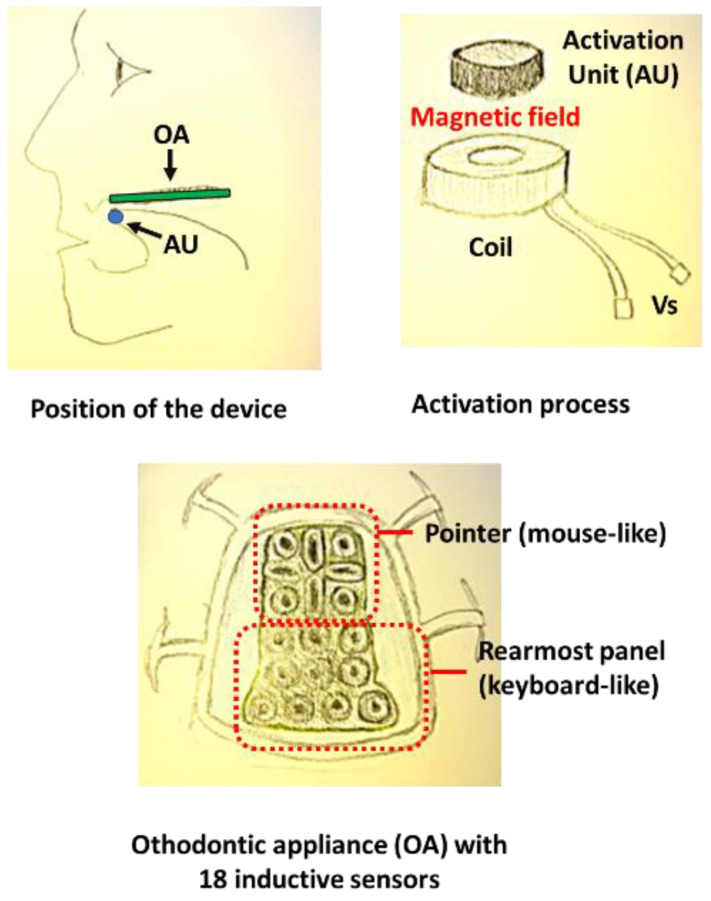
Sketch (realized by D.G.) of the Itongue and its components.

This device, like the previous one, allows persons with severe motor impairments and loss of limb functionality to directly type a text or command a pointing device in order to control, for example, an electric wheelchair or a personal computer. The system consists of an orthodontic appliance (OA) placed on the palate (see the sketch in Figure 4) which contains two blocks of inductive sensors (in total 18) organized in a front panel (8 sensors) that can be used as a pointing device (mouse, joystick) and a rearmost panel (10 sensors) that can function as a keyboard.

Inductive sensors are activated using an activation unit on the OA, which changes the inductance. The activation unit consists of a small cylinder of ferromagnetic material. The activation unit is placed inside the upper sphere of a barbell piercing which is inserted into the tongue and activates a given sensor every time the tongue selects it. The raw activation signals are sent wirelessly to an integrated electronic component (embedded controller) which processes and transforms the data, and sends them to a composite USB peripheral. The USB peripheral then interacts with the PC through a standard USB interface [48]. The high number of sensors and their particular arrangement allows the system to be used both for writing, by the use of each sensor as a key (keyboard mode), and to obtain the functionality of the joystick (mouse mode) combining sensor signals. In addition, the ability of the system to interact with the PC through the USB interface, and therefore without customized software, means that the device can be used with any PC, even one that is not your own. The use of this device gave encouraging results in terms of speed and accuracy from the point of view of the acceptance [39] as described later in the paper (see Section 3.4).

### 3.4. Incorporation in the Health Domain: Acceptance, Safety, Comparison, Regulation Issues

One of the key aspects of the use of these devices is integration into the *health domain*. This integration is based on an accurate assessment of aspects related to interoperability, comparison with the other devices, safety, and the regulations. Targeted searches were carried out on Pubmed, as it is the reference database for the subject of integration into the *health domain*. Other databases and websites (including institutional ones) were also analyzed in order to complement and integrate the available information. Table 4 shows the key search terms used.

#### 3.4.1. Acceptance

The search of studies on acceptance was performed by means of the key in *Position 1* of Table 4. The study reported in [51] analyzed the acceptance of the intraoral inductive tongue system interface in typing text. Both able-bodied and tetraplegic persons were recruited. The experimentation lasted five days. The average error-free typing rate ranged between 11.6 correct characters/min for all participants and 15.5 correct characters/min for those participants familiar with the piercing. The functionality of inductive systems was demonstrated in two quadriplegic subjects and two able-bodied participants in a study reported in [41]. It was particularly appreciated that the device was invisible. A maximum speed of 1.4 s has been reached for the repeated typing of a correct character with the use of the mouse function. The results highlighted both the effectiveness and the aesthetic acceptability of the device. The effectiveness of these devices was also evaluated in an application that involved both the interaction of the TDS with the computer and interaction with the driving systems of an electric wheelchair [52]. Moreover, in this case, both able-bodied participants and participants with spinal cord injuries were recruited [52]. Comparisons were also made with the sip-and-puff (SnP) system, with which patients with spinal cord injuries were already familiar. These comparisons showed that the lingual device obtained three times better speed performance than the SnP. The acceptance of these systems was also investigated in the control of Apps available in the stores for smartphones. A Bluetooth module was used that emulated the interactions of the fingers on the touchscreen [53]. The study was carried out on able-bodied people. The errors were negligible at the typing speed measured.

#### 3.4.2. Comparison

The search of studies on comparison was performed by means of the key in *Position 2* of Table 4. The search returned studies focused on comparison with other systems and on the learning process of the use of these ATs [21,54,55], also highlighting in some cases that these systems could be useful in healthy people to fast control elaborators and mechatronics (e.g., robots). In the study reported in [21], the authors suggested that the inductive tongue control system allowed the user control over the environment. The Cartesian control of an assistive robotic arm was mapped with the device. Two healthy participants were recruited. Trials compared the tongue interface with the manual control based on a keyboard. The results showed that the tongue-based system performed better than the keyboard with an increase in the task duration of up to 30%. The study available in [54] assessed in 18 health participants the ability of the tongue tip to accurately select intraoral targets enclosed in a palatal device. The outcome showed: (a) that the performances were faster and more accurate for targets located farther away from the base of the tongue; (b) an improvement in the speed and accuracy of the learning and familiarity; (c) the evolution of the medical knowledge on the processes of learning related to tongue interaction. Nine able-bodied participants, who already had tongue piercings, were enrolled in another study lasting 5 weeks. The study was focused both on the learning processes on this device and on the limiting factors [55]. Medical knowledge on the human factors affecting the use of these ATs was obtained thanks to the comparison with the index-finger-keypad tools.

#### 3.4.3. Regulation and Safety

The search on regulations with the key in *position 3* of Table 4 did not produce results. This indicates: (a) the need for scholars to focus internationally on these aspects; (b) fragmentation and non-uniformity at an international level, with regard to the applicable regulations. This fragmentation is already present with regard to medical devices, for those with high technological innovation [56]. This causes scarce initiatives and scholarly interest in these issues [56].

The paths for the process of the commercial introduction of these devices, as with the other ones, depend on the country or area (for example, Europe) where they are used, and on the intended use and the relevant application. In fact, there are different legislative frameworks introduced as the country or area changes. These different regulatory frameworks determine different approval and insertion procedures. Thus, the approach, just to give an example, is different if we are in the USA, where the FDA follows a certain road map, than if we are in England, where Nice follows a different approval process, or if we are in Europe where, at the community level, the regulations are determined and must then be implemented in the countries belonging to the community.

These ATs can be used in different applications and with different intended uses. They can be used in clinical or non-clinical applications. They can be used in home automation both to drive electric wheelchairs and to interact generally with the automated home environment. They can be used as a high-tech medical aid to help with expressive language and for communication, or as a part of a mobility or rehabilitation aid. They can be used in a standalone mode or in connection with a network (e.g., local area network, world area network), with different potential implications for cybersecurity.

Regarding safety, AT systems have some requirements to consider relating to the insertion of sensors, actuators (e.g., the barbell piercing), and electronics into the oral cavity.

With these devices being based on electronics, electronic medical safety and electromagnetic compatibility must also be considered. In light of all these considerations, it is evident that a detailed analysis would be impossible and far from the scope of this study given the multifaceted characteristics of the possible applications and their intended uses. With the idea of considering a more general and less specific possible framework, we report the European regulatory framework as an example that is applicable here.

If we focus on the European reality, we find that all the systems in free commerce must follow both the General Product Safety Directive [57] on general product safety and the Directive 85/374/EEC on liability for defective products [58], which together regulate the safety of products on the market and the responsibility for their defects. Furthermore, these AT systems, based on their destination of use, can be classified as a specific medical device (MD). Medical Devices are regulated by (Regulation (EU) 2017/745) [59], which from the year 2020 replaces the previous directive. As electro-medical devices, based on the application, they must comply with the CEI EN 60601-1 standard in force in Europe [60]. As far as electromagnetic compatibility is concerned, the reference standard is Directive 2014/30 /UE EMC [61]. There are three documents that regulate cybersecurity in the EU [62,63,64]:

- The EU Cybersecurity Act (Regulation (EU) 2019/881) which launched an EU certification path dedicated to cybersecurity [62].
- The directive on the security of the network and the information systems (commonly referred to as the NIS Directive) that provides procedures for improving cybersecurity [63].
- The General Data Protection Regulation (GDPR) which necessitates the design of suitable actions to counter cyber risks [64].

The first document made available a procedure for a purely voluntary certification process. The last two documents delegate and pass on security matters to the providers in the *health domain*. 

As for biocompatibility, there is ISO 10993 [65], although it is not mandatory, (this is also applicable in dentistry). It establishes the parameters for the biological evaluation of all medical devices in contact with the human body.

A search on Pubmed, dedicated to safety (*Position* 4, Table 4), highlighted three important studies [46,66,67] in this field. The study reported in [66] investigated the biological consequences of the titanium-magnet tongue implants of the TDS. The authors reported an oromotor and tongue-tissue response in the miniature pig, having a tongue similar to the human tongue. The results suggested the safety of the material used in the implants. Another study addressed this issue [67]. The authors analyzed the behavior of a smooth steel spherical implant in the anterior tongue of the rat. The study showed that the device did not create migration problems or tissue biocompatibility problems. Furthermore, it also showed that tongue functionality was not affected. The study also supports the use of these devices in humans.

In the study reported in [46], the authors designed and tested a tongue-piercing protocol and the application of the magnet barbell piercing to the tongue. They concluded that by using both careful procedures and medical protocols, the risk could be controlled and strongly minimized.

The regulatory framework, with reference to Europe, is of a general nature. It was reported considering the most general and broadest possible cases. It is not certain that, according to the intended use and applications, all regulations must always be applied to these ATs, such as for example what is reported for cybersecurity and electromagnetic compatibility.

Based on the above, an assessment of the marketing status of these devices requires a country-by-country analysis. For the sake of completeness, we report that an inductive operating device is marketed in Europe and is accessible at the site https://tks-technology.dk/en/produkter/#itongue/ (accessed on 7 November 2022) [68].

## 4. Discussion

### 4.1. Highlights from the Study

The study in the first part recalled that: - quadriplegia has an important impact on the autonomy and quality of life of the citizen.-The assistive technologies have both an enormous potential for supporting the communicative functions of people with tetraplegia and are the theme of important studies relating to engineering developments and clinical application. -Among the assistive technologies used in quadriplegia, there are technologies based on the tongue barbell piercing. In the second part of the study it was analyzed the evolution of the tongue piercing as an assistive technology, reporting *four points of view*. The true added values of the study are the *four points of view*.

The *first point of view* made a map point on the evolution of the scientific literature on Pubmed. It reported the scientific production trends. It showed that the scientific production in this sector (see Figure 1) started before the smartphone boom [9] and has not increased, unlike other technologies that the tongue-based systems are capable of controlling, such as the robots used in assistance and rehabilitation. In fact, a search on Pubmed by means of the key “*robotics”[Title/Abstract] AND (“assistance”[Title/Abstract] OR “rehabilitation”[Title/Abstract])*” shows how in the latter sector, the growth up to the year 2021 (the year 2022 is not yet closed) of the scientific production shows a positive pseudo-exponential trend [69]. On the contrary, in the sector of rehabilitation and assistance robots, which these ATs are able to pilot, there has been a disruptive growth of interest. It is therefore important to ask ourselves about the reasons for this disinterest and the factors that influenced it. 

The *second point* of view reported the technological advances and the categorization (see Table 3) according to the position of the scientific literature [8]. These systems have evolved, from time to time, with the evolution of the technologies and processes of miniaturization and have relied on the use of specific microprocessors, FPGAs, protocols based on Bluetooth LTE, interface systems, advanced software (see for example Labview), and typical algorithms of artificial intelligence. 

The *third point of view*, with reference to the barbell piercing, described the two dominant architectural approaches in mouth sensorization (of which, however, there are different evolutions and additions), without the aim of finding the best of the bunch, (it was not the objective of the study). One of these approaches was based on magnetic sensors arranged on orthodontic equipment [32], while another approach was based on palatal equipment with inductive sensors [37]. Even if it was not the objective of the overview to find the best of the bunch, the study suggests that HTA or comparative assessment technology (CAT) studies, focused on the two different systems, could bring a benefit to the development and diffusion of these ATs for the assistance of frail people. Of course, the feasibility of a CAT depends on the availability of both scholars in general and of the two groups responsible for the history of these systems.

The fourth and last *point of view* discussed the integration of these ATs into the *health domain* focusing on the *acceptance, comparison, safety, and* the *regulatory issues*. From one side, the interesting acceptance characteristics of these devices were highlighted, such as:

- The invisibility, which can improve privacy aspects and compliance [41].
- The high typing-speed performances [21,41,51,52,53].
- The better performances than the *SnP* systems in subjects with SCIs [52].
- The ability to improve the use with the increasing time of practice and learning [58,59].
- The effectiveness of control in home automation applications, such as in the control of the electronic wheelchair or of a robotic arm [21,52,53,54].

On the other side, the fragmentation of the legislation at an international level was highlighted. Some key considerations were reported, in some cases with reference to clinical studies [46,66,67] and in other cases with reference to the European regulatory situation [57,58,59,60,61,62,63,64]. It was highlighted that:

- The Pubmed search did not report specific studies on the legislation, useful as an indication for these devices.
- These devices may have different applications and destinations of use. Furthermore, regulatory reference requirements change based on this and according to the country/area of reference.
- Safety covers various aspects (which must be respected in the processes of inclusion in free commerce based on adherence to regulatory requirements) such as, by way of non-exhaustive example and even if not all of these are always applicable: electrical safety and electromagnetic compatibility [60,61]; cybersecurity [62,63,64]; and the biocompatibility of materials [65]. Regarding this last point, in some studies, also based on animal models, scholars have shown how with such systems it is possible to obtain the risk control based on accurate procedures and appropriate medical protocols [46,66,67].

### 4.2. The Tongue-Based Systems versus the Other Assistive Technologies: Pros and Cons

Many technical and psychophysical factors influence the degree of acceptance of a given assistive technology. Among the important factors are the ease of use and the simplicity of learning how the AT works. The device should also be small, discreet, aesthetically acceptable, and of a low cost.

There are currently several AT solutions, with *pros and cons* [5,16].

The *Brain–Computer Interface* (BCI), for example, is a technology based on the ability to read neuronal activity, to process signals, and to send commands to the outside world.

Two approaches are used in the BCI. The first one is Electrocorticography, (ECoG) which is based on intracortical electrodes allowing access to even more intense brain signals. However, these electrodes often cause reactions in the neural tissue in the insertion area. For this reason, the use of these systems is limited to a few cases in which their use is justified, such as in epilepsy. The second approach uses electroencephalography (EEG). In this case, the signals are distant from neurons, resulting in signal attenuation limitations and unreliable performance.

The *Eye Tracker* (ET) captures the movement of the eye and in particular the position of the pupil using a digital camera. This system is influenced by the lighting conditions, sometimes generates eye fatigue, and requires a high level of concentration. There are also alignment problems. In fact, it is necessary, when using it, to maintain the front position with respect to the monitor. False detections may also happen because it is sometimes difficult to distinguish if a point is really of interest for the user or if it is casually pointed.

*Head pointers (HP)* are only suitable for subjects with good residual ability in neck and head movement. They often cause fatigue in the neck and shoulder muscles.

The *Voice recognition systems (VRS)*, dedicated to subjects with an intact ability to speak, are efficient for writing but are slow and not very intuitive when you want to carry out home automation control.

The *SnP system* is one of the most popular ATs. It is based on the pressure value applied to a tube and whether negative or positive pressure is applied (inhalation and exhalation, respectively). This device, although easy, only allows a few direct commands. It also assumes that the user has good control of the diaphragm and airflow, a condition that, for example, limits its use in people who need mechanical ventilation.

These tongue-based systems have shown important potential [8,70,71,72] for wheelchair navigation, computer access, robotic rehabilitation, exoskeleton-based rehabilitation, home automation applications, and in the control of the home environment.

These systems, unlike the tools already in widespread use, seem very flexible and discreet, since they are also able to be hidden [41]. The tongue is connected to the brain by the hypoglossal nerve, which generally escapes serious damage even in spinal cord injuries. The tongue is also the last organ to be affected in most neuromuscular degenerative disorders. It has many degrees of freedom and can move very quickly and accurately within the oral cavity. With the tip of the tongue, you can touch every single tooth, so it is an organ suitable for manipulating this type of auxiliary device. The tongue muscle tires slowly. Therefore, a device based on the tongue motion can be used continuously for long periods, guaranteeing the user a certain degree of privacy, since it remains hidden in the mouth.

These systems: (I) are less invasive than *BCI-ECoG* using intracortical electrodes; (II) can be used on subjects without voice (who cannot use VRSs); (III) can be used on subjects who do not have head movement (who cannot use head pointers); (IV) are totally wearable and invisible; (V) do not need complex alignments as in the case of the *ETs*; and (V) can be used on subjects with displacement problems in the diaphragm (who cannot use the SnP).

However, as highlighted, the paths of adherence to the regulations are complex. The device, with the control electronics, sensors, and an actuator, goes into a cavity of the human body, the oral cavity. This makes the initiatives to be undertaken around regulation certainly more complex than those envisaged for other devices such as HP, VRS, SnP, and ET. Furthermore, the high and complex technology together with the low diffusion increases the costs and engenders a vicious circle that hinders the diffusion of these ATs.

### 4.3. Recommendations from the Study

The WHO is suggesting changing the vision [2] for the better diffusion of ATs, giving great attention to both the needs and acceptance of citizens.

According to the ICDH-2 [2], great attention must be given to the components of health and to the residual capacities of the citizen. The designers of the tongue-based-systems have shown great kindness to persons with tetraplegia through both the improvement and the adaptation of the technology to the various application environments [8,16,17,18,19,20,21], also comprising in some cases the integration of augmented reality and of artificial intelligence [73]. The most commercially available devices are not automatically suitable for everyone as there are different residual capacities, health components, and psychological acceptance aspects to consider in a person.

There is a lack of studies that address the use and applications of these technologies in multiple domains. They must include comparisons, costs, acceptance, dissemination problems, regulatory aspects, ethical aspects, and other issues important for integration into the *health domain*. Studies on health technology assessment, comparative technology assessment, and consensus conferences are therefore now recommended. This would also allow for a better tailoring of the AT devices to the citizen and a wider diffusion of niche devices, such as the ones investigated in this study.

There is therefore the need to invest energy into agreement tools that both support the actors in the *health domain* through recommendations and give a stimulus for stakeholders and researchers. In robotic rehabilitation (a theme that we have seen connected to this topic), for example, the need to face consensus conferences that include experts from various sectors (usually hundreds) was highlighted, and the experiment in progress in Italy that was reported in [74], later concluded in [75]. The issues that should be addressed in a desirable consensus conference for these ATs should include: classification and intended use; clinical and not-clinical use; models of use and research direction: organizational models; training; regulations and ethics.

We hope that this review can be a stimulus for this topic.

### 4.4. Limitations

This study based on a narrative review focused on the integration of these devices into the *health domain* and used Pubmed as the main database, as it is the reference for the interoperability of the devices in the *health domain*. In some search integrations, other databases focused on technologies and websites (including institutional ones) have been used. As far as the regulatory aspects are concerned, we wanted to give a local example, as the search showed that the international situation is fragmented. We referred to the European situation. Further studies are encouraged to expand the regulatory theme to include other local realities.

## 5. Conclusions

This overview was intended to make a map point on the development of ATs based on lingual control with barbell piercing and their integration into the *health domain*.

The overview provides four points of view. The *first point* of view highlights how: (a) the scientific production, which began in this area in the year 2006, has not grown over time; (b) two international research groups have given the greatest impetus in this area. The *second point of view* reported the categorization of these devices together with the relevant elements of technological evolutions. It has been highlighted here that both the emerging innovations of microelectronics and the improvements of the miniaturization processes have been used to achieve the performing results. The *third point of view*, with reference to the use of barbell piercing, reports the two dominant approaches in the device design of the sensorization–activation chain in the mouth. These approaches have been proposed by two international research groups. The *fourth point of view* addresses the aspects of integration into the *health domain*: acceptance, comparison, safety, and the regulatory approach. Important studies and results were highlighted, with concern to performance (three times better than SnP), the learning processes, the high study participant approval of the invisibility of the device, and the biocompatibility and safety studies of the device components. The fragmentation of the legislation on medical devices, for this device, as with all highly innovative technologies, does not help with diffusion. This study highlighted the high potential of these devices. They can also be used by those patients with very low residual capacities, who, for example, cannot use the VRS, HP, or SnP. Stakeholders are advised to invest energy in agreement tools for ATs, such as consensus conferences that allow for, through specific recommendations, the centered and targeted diffusion and use of ATs, including these devices.

## Figures and Tables

**Table 1:** Map between the level of the lesion and the state of neuromuscular functions in quadriplegia.

District/Level	Neuro Respiratory Function	Neuromuscular Function
C1–C4	Need for mechanical breathing	Total paralysis of the arms
C5	Difficulty in coughing, there may be a need for help in cleaning up the secretions	Paralysis of the wrists, hands, and triceps muscles
C6	As above	Paralysis of the wrist flexors, triceps, and hands
C7–C8*	As above	Some muscle weakness in the hand, difficulty in grasping and releasing

**Table 2:** Search terms in Section 3.1.

Search Key
*“tongue drive system”[Title/Abstract] OR “tongue control system”[Title/Abstract] OR “tongue computer”[Title/Abstract] OR “tongue computer interface”[Title/Abstract] OR ((“tongue”[MeSH Terms] OR “tongue”[All Fields] OR “tongues”[All Fields] OR “tongue s”[All Fields]) AND “elaborator”[All Fields] AND (“interface”[All Fields] OR “interface s”[All Fields] OR “interfaced”[All Fields] OR “interfaces”[All Fields] OR “interfacing”[All Fields]))*

**Table 3:** Categorization of the tongue-based systems.

	Young Participants	Brief Description	References
1	Tongue drive system	Actuator and receiver use magnetic fields. Sensors are external	[8,22,23,24,25,26]
2	Intraoral tongue drive system	Actuator and receiver use magnetic fields. Sensors placed inside the mouth.	[8,27,28,29,30,31,32]
3	Standalone tongue drive system	Completely wearable	[8,33,34,35]
4	Multimodal Tongue Drive System	The system integrates a head-mounted device and a speech recognition device	[8,36]
5	Inductive tongue control system	Actuator and receiver (external) use magnetic fields. Inductive sensors are used (placed on a PCB for the palate)	[8,37,38,39,40,41]
6	Further tongue-based systems	They are emerging technologies based on light emitting diodes and light detectors, the IBM tongue track point technologies, piezoelectric sensors, and a tongue-based Joystick (Jouse3)	[8,42,43,44,45]

**Table 4:** The Search Keys in Section 3.4.

	Search Key
1	*(“tongue drive system”[Title/Abstract] OR “tongue control system”[Title/Abstract] OR “tongue computer”[Title/Abstract] OR “tongue computer interface”[Title/Abstract] OR ((“tongue”[MeSH Terms] OR “tongue”[All Fields] OR “tongues”[All Fields] OR “tongue s”[All Fields]) AND “elaborator”[All Fields] AND (“interface”[All Fields] OR “interface s”[All Fields] OR “interfaced”[All Fields] OR “interfaces”[All Fields] OR “interfac-ing”[All Fields]))) AND (“accept”[All Fields] OR “acceptabilities”[All Fields] OR “ac-ceptability”[All Fields] OR “acceptable”[All Fields] OR “acceptably”[All Fields] OR “acceptance”[All Fields] OR “acceptances”[All Fields] OR “acceptation”[All Fields] OR “accepted”[All Fields] OR “accepter”[All Fields] OR “accepters”[All Fields] OR “accepting”[All Fields] OR “accepts”[All Fields])*
2	*(“tongue drive system”[Title/Abstract] OR “tongue control system”[Title/Abstract] OR “tongue computer”[Title/Abstract] OR “tongue computer interface”[Title/Abstract] OR ((“tongue”[MeSH Terms] OR “tongue”[All Fields] OR “tongues”[All Fields] OR “tongue s”[All Fields]) AND “elaborator”[All Fields] AND (“interface”[All Fields] OR “interface s”[All Fields] OR “interfaced”[All Fields] OR “interfaces”[All Fields] OR “interfacing”[All Fields]))) AND (“comparison”[All Fields] OR “comparisons”[All Fields])*
3	*(“tongue drive system”[Title/Abstract] OR “tongue control system”[Title/Abstract] OR “tongue computer”[Title/Abstract] OR “tongue computer interface”[Title/Abstract] OR ((“tongue”[MeSH Terms] OR “tongue”[All Fields] OR “tongues”[All Fields] OR “tongue s”[All Fields]) AND “elaborator”[All Fields] AND (“interface”[All Fields] OR “interface s”[All Fields] OR “interfaced”[All Fields] OR “interfaces”[All Fields] OR “interfacing”[All Fields]))) AND (“legislation and jurisprudence”[MeSH Subheading] OR (“legislation”[All Fields] AND “jurisprudence”[All Fields]) OR “legislation and juris-prudence”[All Fields] OR “regulations”[All Fields] OR “social control, formal”[MeSH Terms] OR (“social”[All Fields] AND “control”[All Fields] AND “formal”[All Fields]) OR “formal social control”[All Fields] OR “regulate”[All Fields] OR “regulates”[All Fields] OR “regulating”[All Fields] OR “regulation s”[All Fields] OR “regulative”[All Fields] OR “regulator”[All Fields] OR “regulator s”[All Fields] OR “regulators”[All Fields] OR “regulated”[All Fields] OR “regulation”[All Fields])*
4	*(“tongue drive system”[Title/Abstract] OR “tongue control system”[Title/Abstract] OR “tongue computer”[Title/Abstract] OR “tongue computer interface”[Title/Abstract] OR ((“tongue”[MeSH Terms] OR “tongue”[All Fields] OR “tongues”[All Fields] OR “tongue s”[All Fields]) AND “elaborator”[All Fields] AND (“interface”[All Fields] OR “interfaces”[All Fields] OR “interfaced”[All Fields] OR “interfaces”[All Fields] OR “interfacing”[All Fields]))) AND (“safety”[MeSH Terms] OR “safety”[All Fields] OR “safeties”[All Fields]))*

## References

[1] Cervical Spinal Cord Injuries. https://www.spinalcord.com/cervical-spinal-cord-injury.

[2] World Health Organization, Assistive Technologies. https://www.who.int/news-room/fact-sheets/detail/assistive-technology.

[3] Assistive Technology Industry Association. https://www.atia.org/home/at-resources/what-is-at/.

[4] ASHA Association. https://www.asha.org/public/speech/disorders/aac/.

[5] Fager S.K., Fried-Oken M., Jakobs T., Beukelman D.R. (2019). New and emerging access technologies for adults with complex communication needs and severe motor impairments: State of the science. Augment. Altern. Commun..

[6] ICDH-2, International Classification of Functioning, Disability and Health. https://unstats.un.org/unsd/disability/pdfs/ac.81-b4.pdf.

[7] Renzoni A., Pirrera A., Lepri A., De Dominicis A., Cammarata P., Meli P., Grigioni M. (2018). Tatuaggi E Piercing: Non Solo Potenziali Rischi, ma Anche Benefici Per I Pazienti. Not Ist Super Sanità. https://www.iss.it/documents/20126/45616/online_aprile_maggio.pdf/7124816f-e1b0-9a7b-96a1-9ab9f8154316?t=1581100961706.

[8] Ghovanloo M., Sahadat M.N., Zhang Z., Kong F., Sebkhi N. (2017). Tapping into tongue motion to substitute or augment upper limbs. Micro-and Nanotechnology Sensors, Systems, and Applications IX.

[9] ANDJ Narrative Checklist. https://it.scribd.com/document/434616519/ANDJ-Narrative-Review-Checklist.

[10] Pubmed Search. https://pubmed.ncbi.nlm.nih.gov/?term=%28%28%28%28tongue+drive+system%5BTitle%2FAbstract%5D%29+OR+%28tongue+control+system%5BTitle%2FAbstract%5D%29%29+OR+%28tongue+computer%BTitle%2FAbstract%5D%29%29+OR+%28tongue+computer+interface%5BTitle%2FAbstract%5D%29+OR+%28tongue+elaborator+interface%29%29&sort=pubdate&size=100.

[11] Giansanti D., Grigioni M. (2018). La Salute in un Palmo di Mano: Nuovi Rischi da Abuso di Tecnologia.

[12] Struijk L.N.S.A. (2006). An Inductive Tongue Computer Interface for Control of Computers and Assistive Devices. IEEE Trans. Biomed. Eng..

[13] Huo X., Wang J., Ghovanloo M. (2007). A Wireless Tongue-Computer Interface Using Stereo Differential Magnetic Field Measurement. Annu. Int. Conf. IEEE Eng. Med. Biol. Soc..

[14] Peng Q., Budinger T.F. (2007). ZigBee-based Wireless Intra-oral Control System for Quadriplegic Patients. Annu. Int. Conf. IEEE Eng. Med. Biol. Soc..

[15] Chou C.-H., Hwang Y.-S., Chen C.-C., Chou S.-W., Chen Y.-L. (2016). Noninvasive tongue-motion controlled computer mouse for the disabled. Technol. Health Care.

[16] Zhang Z., Prilutsky B.I., Butler A.J., Shinohara M., Ghovanloo M. (2021). Design and Preliminary Evaluation of a Tongue-Operated Exoskeleton System for Upper Limb Rehabilitation. Int. J. Environ. Res. Public Health.

[17] Kong F., Sahadat N., Ghovanloo M., Durgin G.D. (2019). A Stand-Alone Intraoral Tongue-Controlled Computer Interface for People With Tetraplegia. IEEE Trans. Biomed. Circuits Syst..

[18] Kim J., Park H., Bruce J., Rowles D., Holbrook J., Nardone B., West D.P., Laumann A., Roth E.J., Ghovanloo M. (2015). Assessment of the Tongue-Drive System Using a Computer, a Smartphone, and a Powered-Wheelchair by People With Tetraplegia. IEEE Trans. Neural Syst. Rehabilitation Eng..

[19] Mohammadi M., Knoche H., Struijk L.N.S.A. (2021). Continuous Tongue Robot Mapping for Paralyzed Individuals Improves the Functional Performance of Tongue-Based Robotic Assistance. IEEE Trans. Biomed. Eng..

[20] Struijk L.N.S.A., Bentsen B., Gaihede M., Lontis R. (2018). Speaking Ability while Using an Inductive Tongue-Computer Interface for Individuals with Tetraplegia: Talking and Driving a Powered Wheelchair-A Case Study. Annu. Int. Conf. IEEE Eng. Med. Biol. Soc..

[21] Struijk L.N.S.A., Lontis R. Comparison of tongue interface with keyboard for control of an assistive robotic arm. Proceedings of the 2017 International Conference on Rehabilitation Robotics (ICORR).

[22] Huo X., Wang J., Ghovanloo M. (2008). Introduction and preliminary evaluation of the tongue drive system: Wireless tongue-operated assistive technology for people with little or no upper-limb function. J. Rehabil. Res. Dev..

[23] Huo X., Wang J., Ghovanloo M. (2008). A Magneto-Inductive Sensor Based Wireless Tongue-Computer Interface. IEEE Trans. Neural Syst. Rehabil. Eng..

[24] Kim J., Huo X., Ghovanloo M. (2010). Wireless control of smartphones with tongue motion using tongue drive assistive technology. Proceedings of the 2010 Annual International Conference of the IEEE Engineering in Medicine and Biology.

[25] Huo X., Wang J., Ghovanloo M. A Wireless Tongue-Computer Interface Using Stereo Differential Magnetic Field Measurement. Proceedings of the 2007 29th Annual International Conference of the IEEE Engineering in Medicine and Biology Society.

[26] Huo X., Wang J., Ghovanloo M. A Magnetic Wireless Tongue-Computer Interface. Proceedings of the 2007 3rd International IEEE/EMBS Conference on Neural Engineering.

[27] Tang H., Beebe D.J. (2006). An oral tactile interface for blind navigation. IEEE Trans. Neural Syst. Rehabil. Eng..

[28] Randerath W.J., Galetke W., Domanski U., Weitkunat R., Ruhle K.-H. (2004). Tongue-muscle training by intraoral electrical neurostimulation in patients with obstructive sleep apnea. Sleep.

[29] Campisi G., Giannola L.I., Florena A.M., De Caro V., Schumacher A., Göttsche T., Paderni C., Wolff A. (2010). Bioavailability in vivo of naltrexone following transbuccal administration by an electronically-controlled intraoral device: A trial on pigs. J. Control. Release.

[30] Park H., Ghovanloo M. (2016). A Wireless Intraoral Tongue–Computer Interface. Wirel. Med. Syst. Algorithms: Des. Appl..

[31] Park H., Kiani M., Lee H.-M., Kim J., Block J., Gosselin B., Ghovanloo M. (2012). A Wireless Magnetoresistive Sensing System for an Intraoral Tongue-Computer Interface. IEEE Trans. Biomed. Circuits Syst..

[32] Park H., Ghovanloo M. (2014). An Arch-Shaped Intraoral Tongue Drive System with Built-in Tongue-Computer Interfacing SoC. Sensors.

[33] Yousefi B., Huo X., Kim J., Veledar E., Ghovanloo M. (2012). Quantitative and Comparative Assessment of Learning in a Tongue-Operated Computer Input Device–-Part II: Navigation Tasks. IEEE Trans. Inf. Technol. Biomed..

[34] Viseh S., Ghovanloo M., Mohsenin T. (2015). Toward an Ultralow-Power Onboard Processor for Tongue Drive System. IEEE Trans. Circuits Syst. II Express Briefs.

[35] Jafari A., Buswell N., Page A., Mohsenin T., Sahadat M.N., Ghovanloo M. Live demonstration: Towards an ultra low power on-board processor for Tongue Drive System. Proceedings of the 2015 IEEE Biomedical Circuits and Systems Conference (BioCAS).

[36] Sahadat M., Alreja A., Srikrishnan P., Ghovanloo M. A multimodal human computer interface combining head movement, speech and tongue motion for people with severe disabilities. Proceedings of the 2015 IEEE Biomedical Circuits and Systems Conference (BioCAS).

[37] Struijk L.N. (2006). A tongue based control for disabled people. International Conference on Computers for Handicapped Persons.

[38] Struijk L.N.S.A., Lontis E.R., Bentsen B., Christensen H.V., Caltenco H.A., Lund M.E. (2009). Fully integrated wireless inductive tongue computer interface for disabled people. 2009 Annual International Conference of the IEEE Engineering in Medicine and Biology Society.

[39] Lontis E.R., Lund M.E., Christensen H.V., Bentsen B., Gaihede M., Caltenco H.A., Struijk L.N.S.A. Clinical evaluation of wireless inductive tongue computer interface for control of computers and assistive devices. Proceedings of the 2010 Annual International Conference of the IEEE Engineering in Medicine and Biology.

[40] Lund M.E., Christiensen H.V., Caltenco H.A., Lontis E.R., Bentsen B., Struijk L.N.A. Inductive tongue control of powered wheelchairs. Proceedings of the 2010 Annual International Conference of the IEEE Engineering in Medicine and Biology.

[41] Struijk L.N.S.A., Lontis E.R., Gaihede M., Caltenco H.A., Lund M.E., Schioeler H., Bentsen B. (2016). Development and functional demonstration of a wireless intraoral inductive tongue computer interface for severely disabled persons. Disabil. Rehabil. Assist. Technol..

[42] Saponas T., Scott D., Kelly B.A., Tan D.S. (2009). Optically sensing tongue gestures for computer input. Proceedings of the 22nd Annual ACM Symposium on User Interface Software and Technology.

[43] Salem C., Zhai S. (1997). An isometric tongue pointing device. Proceedings of the ACM SIGCHI Conference on Human Factors in Computing Systems.

[44] Nutt W., Arlanch C., Nigg S., Staufert G. (1998). Tongue-mouse for quadriplegics. J. Micromech. Microeng..

[45] Jouse3. Compusult Limited. http://www.jouse.com/.

[46] Laumann A., Holbrook J., Minocha J., Rowles D., Nardone B., West D., Kim J., Bruce J., Roth E., Ghovanloo M. (2015). Safety and Efficacy of Medically Performed Tongue Piercing in People with Tetraplegia for Use with Tongue Operated Assistive Technology. Top Spinal. Cord Inj. Rehabil..

[47] Minocha J.S., Holbrook J.S., West D.P., Ghovanloo M., Laumann A.E. (2014). Development of a Tongue-Piercing Method for Use With Assistive Technology. JAMA Dermatol..

[48] Lontis E.R., Struijk L.N. (2010). Design of inductive sensors for tongue control system for computers and assistive devices. Disabil. Rehabil. Assist. Technol..

[49] Bentsen B., Gaihede M., Lontis R., Struijk L.N.A. (2014). Medical tongue piercing – development and evaluation of a surgical protocol and the perception of procedural discomfort of the participants. J. Neuroeng. Rehabil..

[50] Lund M.E., Caltenco H.A., Lontis E.R., Christiensen H.V., Bentsen B., Struijk L.N.S.A. A framework for mouse and keyboard emulation in a tongue control system. Proceedings of the 2009 Annual International Conference of the IEEE Engineering in Medicine and Biology Society.

[51] Struijk L.N.S.A., Bentsen B., Gaihede M., Lontis E.R. (2017). Error-Free Text Typing Performance of an Inductive Intra-Oral Tongue Computer Interface for Severely Disabled Individuals. IEEE Trans. Neural Syst. Rehabil. Eng..

[52] Kim J., Park H., Bruce J., Sutton E., Rowles D., Pucci D., Holbrook J., Minocha J., Nardone B., West D. (2013). The Tongue Enables Computer and Wheelchair Control for People with Spinal Cord Injury. Sci. Transl. Med..

[53] Kim J., Park H., Ghovanloo M. Tongue-operated assistive technology with access to common smartphone applications via Bluetooth link. Proceedings of the 2012 Annual International Conference of the IEEE Engineering in Medicine and Biology Society.

[54] Caltenco H.A., Lontis E.R., Boudreau S.A., Bentsen B., Struijk J., Struijk L.N.S.A. (2011). Tip of the Tongue Selectivity and Motor Learning in the Palatal Area. IEEE Trans. Biomed. Eng..

[55] Yousefi B., Huo X., Veledar E., Ghovanloo M. (2011). Quantitative and Comparative Assessment of Learning in a Tongue-Operated Computer Input Device. IEEE Trans. Inf. Technol. Biomed..

[56] Giansanti D. (2022). The Regulation of Artificial Intelligence in Digital Radiology in the Scientific Literature: A Narrative Review of Reviews. Healthcare.

[57] (2001). Directive 2001/95/EC of the European Parliament and of the Council of 3 December 2001 on General Product Safety. https://eur-lex.europa.eu/legal-content/EN/TXT/?uri=celex%3A52003PC0048.

[58] Directive 85/374/EEC-Liability for Defective Products. https://osha.europa.eu/it/legislation/directives/council-directive-85-374-eec.

[59] (2017). Regulation (EU) 2017/745 of the European Parliament and of the Council of 5 April 2017 on Medical Devices, Amending Directive 2001/83/EC, Regulation (EC) No 178/2002 and Regulation (EC) No 1223/2009 and Repealing Council Directives 90/385/EEC and 93/42/EEC. https://eur-lex.europa.eu/legal-content/EN/TXT/HTML/?uri=CELEX:32017R0745&from=IT.

[60] IEC 60601-1 Edition 3.1 2012-08. INTERNATIONAL STANDARD. http://www.pacificcrn.com/Upload/file/201705/06/20170506193715_57243.pdf.

[61] DIRECTIVE 2014/30/EU of the European Parliament and of the Council of 26 February 2014 on the Harmonisation of the Laws of the Member States Relating to Electromagnetic Compatibility. https://eur-lex.europa.eu/legal-content/EN/TXT/PDF/?uri=CELEX:32014L0030&rid=4#:~:text=This%20Directive%20regulates%20the%20electromagnetic,adequate%20level%20of%20electromagnetic%20compatibility.

[62] Shaping Europe’s Digital Future. https://digital-strategy.ec.europa.eu/en/policies/cybersecurity-act.

[63] NIS Directive (The Directive on Security of Network and Information Systems). https://www.itgovernance.eu/fi-fi/nis-directive-fi.

[64] Complete Guide to GDPR Compliance. https://gdpr.eu/.

[65] ISO 10993-23:2021 Biological Evaluation of Medical Devices. https://www.iso.org/standard/74151.html.

[66] Sokoloff A.J., Yang Z., Sargolzaei S., Strait K., Krasnopeyev A., Easley K.A., Mimche S., Ghovanloo M. (2017). Magnetic implants in the tongue for assistive technologies: Tests of migration; oromotor function; and tissue response in miniature pigs. Arch. Oral Biol..

[67] Mimche S., Ahn D., Kiani M., Elahi H., Murray K., Easley K., Sokoloff A., Ghovanloo M. (2016). Tongue implant for assistive technologies: Test of migration, tissue reactivity and impact on tongue function. Arch. Oral Biol..

[68] Itongue, Communication and Wheelchair Controller in One Product. https://tks-technology.dk/en/produkter/#itongue.

[69] Pubmed Search. https://pubmed.ncbi.nlm.nih.gov/?term=%28robotics%5BTitle%FAbstract%5D%29+AND+%28%28+assistance%5BTitle%2FAbstract%5D%29+OR+%28rehabilitation%5BTitle%FAbstract%5D%29%29&sort=date&size=200.

[70] Palsdottir A.A., Mohammadi M., Bentsen B., Struijk L.N.S.A. (2022). A Dedicated Tool Frame Based Tongue Interface Layout Improves 2D Visual Guided Control of an Assistive Robotic Manipulator: A Design Parameter for Tele-Applications. IEEE Sensors J..

[71] Thøgersen M.B., Mohammadi M., Gull M.A., Bengtson S.H., Kobbelgaard F.V., Bentsen B., Ali Khan B.A., Severinsen K.E., Bai S., Bak T. (2022). User Based Development and Test of the EXOTIC Exoskeleton: Empowering Individuals with Tetraplegia Using a Compact, Versatile, 5-DoF Upper Limb Exoskeleton Controlled through Intelligent Semi-Automated Shared Tongue Control. Sensors.

[72] Lontis E.R., Bentsen B., Gaihede M., Biering-Sørensen F., Struijk L.N.A. (2021). Wheelchair Control with Inductive Intra-Oral Tongue Interface for Individuals with Tetraplegia. IEEE Sens. J..

[73] Chu F.J., Xu R., Zhang Z., Vela P.A., Ghovanloo M. Vela, and Maysam Ghovanloo The Helping Hand: An Assistive Manipulation Framework Using Augmented Reality and Tongue-Drive Interfaces. Proceedings of the 2018 40th Annual International Conference of the IEEE Engineering in Medicine and Biology Society (EMBC).

[74] Maccioni G., Ruscitto S., Gulino R.A., Giansanti D. (2021). Opportunities and Problems of the Consensus Conferences in the *Care Robotics*. Healthcare.

[75] Consensus Conference Cicerone, Document Finale. https://www.simfer.it/consensus-conference-cicerone-documento-finale-conclusivo/.

